# Edson’s New Ear Trumpets

**Published:** 1878-10

**Authors:** 


EDISON'S NEW EAR TRUMPETS.—The "Scientific American" says that Professor Edison, in his researches on sound, has made many curious experiments, one of the most interesting of which is that of conversing through a distance of 1½ to 2 miles, with no other apparatus than a few paper funnels. These funnels constitute the megaphone, an instrument wonderful both for its simplicity and effectiveness. Such an instrument stands on the balcony of Professor Edison's laboratory. A mile and a half distant there is another instrument exactly like the first one. The two larger funnels are 6 feet 8 inches long, and 27½ inches in diameter at the larger end. These funnels are each provided with a flexible ear tube, the end of which is placed in the ear. The speaking trumpet in the middle does not differ materially from the ordinary ones. It is a little longer and has a larger bell mouth. With this instrument conversation can be readily carried on through a distance of 1½ to 2 miles. A low whisper, uttered without using the speaking trumpet, is distinctly audible at a thousand feet, and walking through grass and weeds may be heard at a much greater distance. —*American Med. Bi-Weekly.*

